# The Oral NOAEL of Flurochloridone in Male Wistar Rats in Ninety-Day Subchronic Toxicity Test Was 3mg/kg/day

**DOI:** 10.3390/ijerph16040553

**Published:** 2019-02-14

**Authors:** Hongyan Zhu, Rui Li, Su Zhou, Suhui Zhang, Yu Wang, Shihong Liu, Qingwen Song, Xiuli Chang, Yubin Zhang, Luqing Liu, Liming Tang, Zhijun Zhou

**Affiliations:** 1 Pharmacology and Toxicology Department, Shanghai Institute for Food and Drug Control, Shanghai 201203, China; zhuhy-0@126.com (H.Z.); lirui@sifdc.org.cn (R.L.); zhousu@sifdc.org.cn (S.Z.); suhuizhang76@163.com (S.Z.); wangyu@sifdc.org.cn (Y.W.); 2 School of Public Health/MOE Key Laboratory for Public Health Safety/Collaborative Innovation Center of Social Risks Governance in Health, Fudan University, Shanghai 200032, China; 1990.shi.hong@163.com (S.L.); 13211020017@fudan.edu.cn (Q.S.); xlchang@shmu.edu.cn (X.C.); yz001@fudan.edu.cn (Y.Z.); lqliu15@fudan.edu.cn (L.L.)

**Keywords:** Flurochloridone, subchronic toxicity, NOAEL, male reproductive system

## Abstract

A ninety-day toxicity and toxicokinetics of flurochloridone (FLC) were studied in male Wistar rats with oral administration at doses of 3 mg/kg and 10 mg/kg respectively, following the previous study. Apparent toxicity to reproductive system of male rats was still observed at the dose of 10 mg/kg, trace amounts of FLC were still detected 24 hours after administration, testicular weight, epididymal weight and serum testosterone were significantly reduced and sperm abnormalities in epididymis were significantly increased. No abnormalities were found in 3 mg/kg group, it indicated that no-observed-adverse-effect level (NOAEL) of FLC in male rats was 3 mg/kg/day, far below the dose of 20 mg/kg/day reported by European Food Safety Authority (EFSA). Therefore, more attention should be paid to this herbicide.

## 1. Introduction

Flurochloridone (FLC) is a pyrrolidone herbicide developed by Stauffer Chemical CO. in 1970s and put into production in 1980s. FLC is effective against a wide variety of broadleaf weeds. It is widely used in Serbia [1], Argentina [2], and Turkey [3]. Research showed that FLC had an excellent prospect for weed control in garlic fields in China [4], this may lead to a gradual increase in FLC usage.

According to the European Food Safety Authority (EFSA)’s report, acute and short-term no-observed-adverse-effect levels (NOAELs) of FLC were 20 mg/kg/day in rats, where the lowest-observed-adverse-effect level (LOAEL) was 25 mg/kg/day [5]. Our previous study showed that testis and epididymis were the organs that were injured the most seriously after 90-day administration, and at the dose of 31.25 mg/kg, FLC caused severe damages to testes and epididymides of male rats but had no adverse effect on female rats [6]. Considering the wide application of FLC in the world, it is reasonable to assess its risk to male continuously. Therefore, 3 mg/kg and 10 mg/kg were set into a subchronic toxicity study in male rats to explore NOAEL and provide more powerful evidence for health risk assessment.

## 2. Materials and Methods

### 2.1. Materials

Flurochloridone (3-chloro-4-(chloromethyl)-1-(3-(trifluoromethyl)phenyl)-2-pyrrolidinone, PubChem CID: 91677) (purity >95.5%) was purchased from Jiangxi Anlida Chemical Co., Ltd. (Jiangxi, China). FLC was suspended in 0.5% (w/v) sodium carboxymethyl cellulose (CMC-Na) used as a vehicle at concentrations of 0.15 mg/mL and 0.5 mg/mL, and fresh samples were prepared every three days. The suspension was stirring during oral administration at room temperature.

A FLC standard (purity 99.0%) was purchased from Sigma-Aldrich (Seelze, Germany). Verapamil was provided by Shanghai Institute for Food and Drug Control (SIFDC, Shanghai, China) as an internal standard for Ultra Performance Liquid Chromatography-Tandem Mass Spectrometry UPLC-MS/MS analysis of FLC.

Levels of follicle-stimulating hormone (FSH), luteinizing hormone (LH), and testosterone in serum were measured by radioimmunoassay with immunoassay kit from Beijing North Institute of Biological Technology.

### 2.2. Experimental Animals and Housing Conditions

This study was conducted at SIFDC with all protocols approved by the Institutional Animal Care and Use Committee (IACUC) of SIFDC (IACUC-SIFDC16106 date: 21/09/16). Six-week-old male Wistar rats were obtained from Shanghai SLAC Laboratory Animal Co., Ltd. (Shanghai, China). Animals were kept in a room maintained at 23 ± 2℃, relative humidity of 40%–70%, under a 12 h light/dark cycle. All rats were fed a standard diet for one week to adapt to the environment.

Clinical observations were performed every day for general conditions of all animals, including hair color, behavior, respiration, secretion, feeding, excretion, and so on. Body weight and food consumption data were collected once a week during study period.

### 2.3. Subchronic Oral Toxicity Study

Eighteen male rats were randomly divided into three groups according to body weight. Control group were administrated orally on daily basis for ninety days (weeks 1–13) with 0.5% (w/v) CMC-Na, while 3 mg/kg group and 10 mg/kg group received a corresponding dose of FLC, respectively, and the dosage was adjusted according to body weight after each weighing.

#### 2.3.1. Toxicokinetic Evaluation in Rats

The blood of rats for toxicokinetic evaluation in each group were sampled at 0.5, 1, 2, 3, 5, 8, 10, and 24 hours after gavage at first day, 30, 60, and 90 days, respectively. Plasma FLC concentration was determined as previously described [6,7].

#### 2.3.2. Necropsy and Histopathology

On the last day, rats in each group were sacrificed for necropsy. Animals’ blood were drawn via the abdominal aorta, then testes and epididymides were separated, and left epididymal tail was removed, placed in a 37℃ water bath, cut with scissors, and incubated for 3–5 mins to allow the sperms to swim out, and diluted for sperm abnormality examination. Then testes and epididymides were put into modified Davison’s fixative for 24 hours and then transferred into 10% neutral buffered formalin for histopathology. Serum was accumulated by centrifugation at 804× *g* for 10 min at 4 °C and then determined levels of FSH, LH, and testosterone by radioimmunoassay.

### 2.4. Statistical Analysis

All measurements were expressed as the means ± standard deviation. Statistical analysis was performed with the *t*-test using SPSS software (IBM SPSS Statistics Version 19.0, Chicago, IL, USA). *p*-values lower than 0.05 were considered to be significant. Toxicokinetic parameters (T_max_, C_max_, Clearance [CLz/F] and area under the curve [AUC]) in rats were assessed by a non-compartmental method using the Drug and Statistics 2.1 (DAS 2.1) software package (Mathematical Pharmacology Professional Committee of China, Shanghai, China).

## 3. Results

### 3.1. Clinical Observation

**Figure 1:**
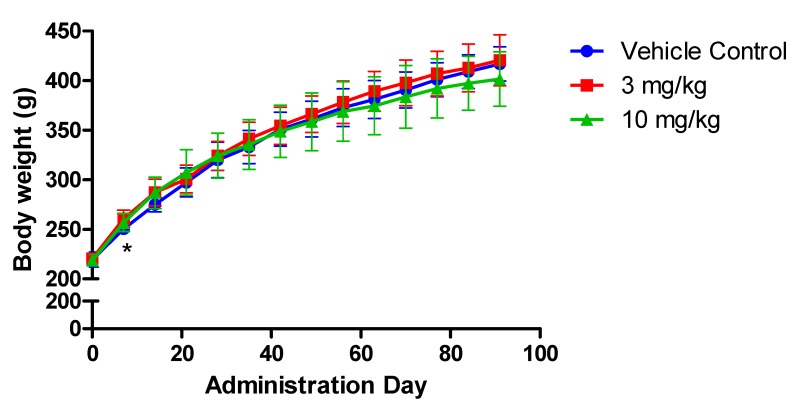
Mean body weight of rats treated with flurochloridone (FLC) for ninety days. * A significant difference at *p* < 0.05 level compared with the control.

No abnormalities were observed during clinical observation. Body weight in two FLC-treated groups increased time dependently. For the 3 mg/kg group, the weight was significantly increased compared with vehicle control group on administration day 7 (*p* < 0.05) (Figure 1). Although the statistically significance was observed, the biological relevance was not indicated during the continual observation on the body weight in this group.

### 3.2. Toxicokinetic Evaluation in Rats

**Figure 2:**
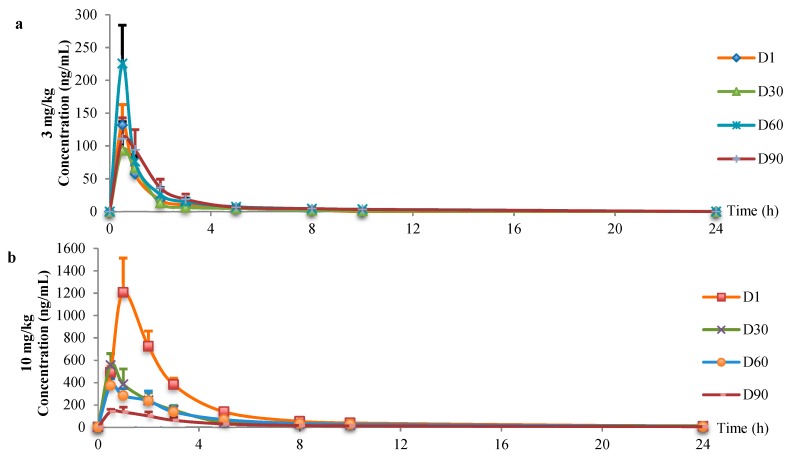
Mean plasma concentration-time profiles. (a) 3 mg/kg. (b) 10 mg/kg.

Mean plasma concentration-time profiles were shown in Figure 2. The FLC plasma concerntration in the 3 mg/kg group was gradually decreased after oral administration and completely metabolized after 24 hours with no FLC detected. Rats in the 10 mg/kg group could still detect trace amounts of FLC after 24 hours. Toxicokinetic parameters of FLC in rats were shown in Table 1.

Toxicokinetic parameters were irregular in 3 mg/kg group. AUC_(0-t)_ varied between 174.95 and 456.18 μg·L^−1^·h, clearance varied between 6.34 and 17.93 L·h^−1^·kg^−1^. In 10 mg/kg group, AUC_(0-t)_ gradually decreased (from 3019.81 to 609.05 μg·L^−1^·h) and clearance gradually increased (from 3.33 to 16.71 L·h^−1^·kg^−1^) with the prolongation of administration.

### 3.3. Endocrine Function

Serum testosterone levels were significantly reduced in rats with FLC at 10 mg/kg (*p* < 0.05) while no significant difference was found in serum FSH and LH levels (*p* > 0.05) (Table 2).

### 3.4. Necropsy and Histopathology

No macroscopic abnormalities were found during necropsy, and organ coefficients of epididymis and testis were shown in Table 3. There had been a significant decrease in testis and epididymis in 10 mg/kg group. Sperm abnormality rate at 10 mg/kg group was significantly higher (*p* < 0.01) than that of the solvent control group, there was no significant difference between the 3 mg/kg group and the solvent control group (Table 3).

**Figure 3:**
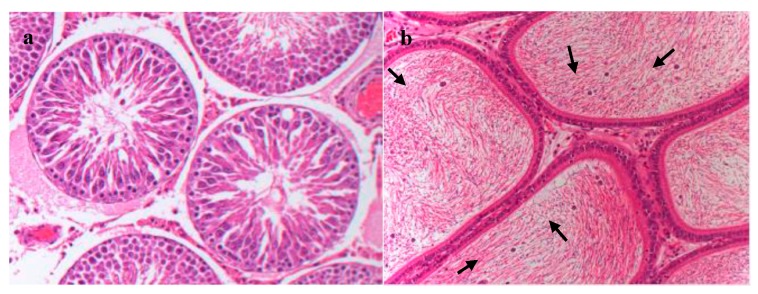
Histopathology of the testis and epididymis of rats treated with 10 mg/kg FLC for ninety days (at 200×magnification, H&E stain). (**a**) Testis. (**b**) Epididymis. Note: Black arrows represent cell debris.

Histopathological examination showed that after FLC administrated for ninety days, the structure of testis and epididymis in the 3 mg/kg group was similar to that of the solvent control group, with no obvious abnormalities. No significant histopathological changes were found in the testicular structure of 10 mg/kg group, cell debris were present occasionally in the epididymal lumen (Figure 3).

## 4. Discussion

Flurochloridone was included in Annex I to Directive 91/414/EEC as an herbicide on 1 June 2011 by the European Union (EU). According to the EU’s requirements, re-evaluation work was needed to determine its impact on human health and environment. Because there are no data on population exposure, our FLC’s experimental data on rats are very important. Our previous study [6] found that rats’ testes and epididymides were seriously damaged by FLC at doses of 31.25 mg/kg, 125 mg/kg and 500 mg/kg, and NOAEL was not found. As a subsequent study, two doses of FLC (3 mg/kg and 10 mg/kg) were tested. FLC had a significant effect on various parameters of rats at the doses of 10 mg/kg, including decreased testicular weight, epididymal weight and serum testosterone level, caused cell debris in the epididymal lumen and increased sperm abnormality rate while no abnormalities were found in any parameters in 3 mg/kg group. This study and our previous study [6] showed a good dose-response relationship between dosage from 3 mg/kg to 500 mg/kg with the adverse effect from no damage to severe injury. So the oral NOAEL of FLC in male rats in 90-day toxicity was 3 mg/kg/day, far below the dose of 20 mg/kg/day reported by EFSA.

Toxicokinetic evaluation revealed that 3 mg/kg of FLC can be excreted by rats in a single day, FLC is completely metabolized and could not be detected with irregular toxicokinetic parameters 24 hours after administration. With the time of repeated administration reached ninety days, 3 mg/kg of FLC could still be completely excreted by rats in a single day. In the 10 mg/kg group, trace amounts of FLC were still detected after 24 hours. All these were in accordance with toxic effects. 

After 90 days administration of FLC, no significant changes in rats’ behavior and body weight were observed. While the testicular and epididymal weight lost in the 10mg/kg group. Body weight and internal organ weight were the main parameters used in the assessment of toxicity [8,9], this indicated that FLC was toxic to testes and epididymides. The decreased weight of testes may be attributed to a decrease in sperm production, as the weight depended on the number of differentiated spermatogenic cell [10]. Although histopathological examination did not reveal any change in testes, cell debris was present occasionally in the epididymal lumen of the 10 mg/kg group. We presumed that the cell debris in epididymis may be due to the premature release of immature sperm cells.

Spermatogenesis is more sensitive to environmental contaminants and significantly influenced compared to their female counterparts [11], so sperm analysis is a sensitive indicator of male reproduction function. The effect of environment toxicants on sperm quality could be judged by sperm morphology and quantity [12]. We found that FLC caused a significant increase in sperm abnormality in epididymis at the dose of 10 mg/kg, which was consistent with the histopathological findings of epididymis. These results revealed that FLC can affect spermatogenesis, resulting in reduced spermatogenic cells in the spermatogenic epithelium and increased sperm abnormalities, eventually resulting in decreased testicular and epididymal weight at the dose of 10 mg/kg.

Testosterone is the main product of gonadal steroid synthesis and plays an important role in the initiation of spermatogenesis and sperm maturation [13]. It is regulated by the endocrine system, affected by LH and FSH, and was synthetized in Leydig cells [14]. LH and FSH can also maintain the normal structure and function of testis [15] by regulating Sertoli cell function [16]. In this study, FLC exposure caused a dose-dependent decrease in serum testosterone levels, with no significant changes in LH and FSH levels compared with the solvent control group. So FLC may affect the level of Testosterone by other ways rather than affecting secretion of LH and FSH. And from these results in our study, we speculated that decreased testosterone level affected spermatogenesis process, and led to a decrease in testicular and epididymal weight.

The increase of male infertility is closely related to these chemical pollutants [17], such as PBDEs [18] and atrazine [19], they are affecting our health all the time. The effects of FLC on male reproductive system will ultimately affect human beings through the accumulation of biological chains. The NOAEL we had obtained was an order of magnitude lower than that reported by EFSA, for this reason, we need to pay more attention to the risk assessment of FLC until further population exposure and health data are available. In addition, considering that FLC is highly targeted to testis and has almost no damage to tissues other than testes at the same dose, the development of FLC in testicular targeting drugs can be considered.

## 5. Conclusions

The present ninety-day toxicity study demonstrated that FLC had obvious toxicity to male Wistar rats at the dose of 10 mg/kg, trace amounts of FLC were still detected 24 hours after administration, testicular weight, epididymal weight and serum testosterone were significantly reduced and sperm abnormalities in epididymis were significantly increased. No abnormalities were found at the dose of 3 mg/kg. Based on the results, the NOAEL for FLC in male rats was estimated to be 3 mg/kg/day in present study, far below the dose of 20 mg/kg/day reported by EFSA.

## Figures and Tables

**Table 1:** Toxicokinetic parameters of FLC (Mean ± SD).

Group	Parameters	Day 1	Day 30	Day 60	Day 90
3 mg/kg	AUC_(0-t)_ (μg·L^−1^·h)	254.11 ± 63.93	174.95 ± 46.28	456.18 ± 118.38	278.57 ± 56.14
	T_max_ (h)	0.50 ± 0.00	0.60 ± 0.22	0.50 ± 0.00	0.60 ± 0.22
	CLz (L·h^−1^·kg^−1^)	11.97 ± 2.88	17.93 ± 3.75	6.34 ± 1.25	11.25 ± 3.00
	C_max_ (μg/L)	131.80 ± 31.14	99.19 ± 36.01	225.60 ± 58.58	113.94 ± 32.19
10 mg/kg	AUC_(0-t)_ (μg·L^−1^·h)	3019.81 ± 488.96	1469.59 ± 410.57	1453.90 ± 283.36	609.05 ± 201.43
	T_max_ (h)	1.00 ± 0.00	0.50 ± 0.00	0.50 ± 0.00	0.70 ± 0.27
	CLz (L·h^−1^·kg^−1^)	3.33 ± 0.63	7.14 ± 2.03	6.91 ± 1.08	16.71 ± 4.24
	C_max_ (μg/L)	1206.20 ± 307.84	579.00 ± 102.67	372.80 ± 64.32	156.20 ± 33.96

Note: SD—standard deviation.

**Table 2:** Summary of hormone levels in rats (Mean ± SD).

Parameters	Control	3 mg/kg	10 mg/kg
FSH (mIU/ml)	3.09 ± 1.19	3.77 ± 0.82	3.16 ± 0.21
LH (mIU/ml)	4.75 ± 0.39	5.01 ± 1.24	4.47 ± 0.82
Testosterone (ng/ml)	1.01 ± 0.31	0.56 ± 0.34	0.17 ± 0.07 ^*^

Note: * A significant difference at *p* < 0.05 level compared with the control. FSH—follicle-stimulating hormone; LH—luteinizing hormone.

**Table 3:** Summary of necropsy (Mean ± SD).

Parameters	Control	3 mg/kg	10 mg/kg
Epididymis ratio	0.2892 ± 0.0661	0.2626 ± 0.0195	0.2313 ± 0.0196 ^*^
Testis ratio	0.9176 ± 0.1526	0.8275 ± 0.0066	0.7642 ± 0.0600 ^*^
Sperm abnormality rate (%)	0.47 ± 0.19	0.51 ± 0.26	1.14 ± 0.38 ^**^

Note: * A significant difference at *p* < 0.05 level compared with the control. ^**^ A significant difference at *p* < 0.01 level compared with the control.
